# Enhanced Flexibility of Biodegradable Polylactic Acid/Starch Blends Using Epoxidized Palm Oil as Plasticizer

**DOI:** 10.3390/polym10090977

**Published:** 2018-09-02

**Authors:** Raina Jama Awale, Fathilah Binti Ali, Azlin Suhaida Azmi, Noor Illi Mohamad Puad, Hazleen Anuar, Azman Hassan

**Affiliations:** 1 Department of Biotechnology Engineering, Kulliyyah of Engineering, International Islamic University Malaysia (IIUM), Jalan Gombak, 53100 Kuala Lumpur, Malaysia; raina.awale@gmail.com (R.J.A.); azlinsu76@iium.edu.my (A.S.A.); illi@iium.edu.my (N.I.M.P.); 2 Department of Manufacturing and Materials Engineering, Kulliyyah of Engineering, International Islamic University Malaysia (IIUM), Jalan Gombak, 53100 Kuala Lumpur, Malaysia; hazleen@iium.edu.my; 3 Faculty of Engineering, Universiti Teknologi Malaysia (UTM), 81310 Johor Bahru, Malaysia; azmanh@cheme.utm.my

**Keywords:** polylactic acid, biodegradable, starch, epoxidized palm oil, solution casting, green plasticizer

## Abstract

The brittleness of polylactic acid (PLA) has always limited its usage, although it has good mechanical strength. In this study, flexibility of PLA/starch (PSt) blend was enhanced using epoxidized palm oil (EPO) as the green plasticizer. The PLA/starch/EPO (PSE) blends were prepared while using the solution casting method by fixing the content of starch and varying ratio of EPO. The thermal properties, such as glass transition temperature (*T_g_*), melting temperature (*T_m_*), and crystallization temperature (*T_cc_*) were decreased by increasing the amount of EPO into PSt, indicating that EPO increases the chain mobility. Thermogravimetric analysis (TGA) showed that thermal degradation resistance of PSE was higher when compared to PSt. The mechanical testing revealed that EPO at all contents improved the mechanical properties, such as increment of the elongation-at-break and impact strength. Whereas, dynamic mechanical analysis showed that the addition of filler into PLA decreased the storage modulus of PLA. The carbonyl group of the aliphatic ester remained the same in the PSE blends. The morphological study verified the ductility of PSE blends surface when compared to the brittle surface of PSt. As for the soil burial tests, EPO accelerated the degradation of blends. From these results, it can be concluded that EPO improved the flexibility of PLA blends.

## 1. Introduction

Plastics, which have always been used in the packaging industry, are mostly prepared from petroleum-based materials. Among their attractions are durability and stability of plastics to external biotic and abiotic stresses [1]. However, the excellent durability of the petroleum-based plastics makes it to be non-degradable. Thus, it raises concern on the environmental pollution caused by accumulation of plastic waste in the landfill. For this purpose, biodegradable and sustainable resources of materials are needed as an alternative to the commodity plastics.

Considerable efforts have been devoted by many researchers to develop biodegradable polymer from renewable resources [2]. One of the bio-based polymers that are suitable for biodegradable packaging material is polylactic acid (PLA). PLA is a linear aliphatic polyester that is polymerized using its monomer, which is lactic acid. Lactic acid can be produced via the fermentation of starch, corn, rice, or sugarcane bagasse. The distinctive features of PLA, such as processability, biocompatibility, and environmentally friendly favored its applications in textile, medical, and as a packaging material [3,4,5]. However, high cost, low flexibility, hydrophobicity, and slow degradation rate of PLA have limited its applications.

In order to reduce the cost and improve the flexibility of PLA, it has been modified by blending with low cost fillers, such as fiber [6], wood flour [7], and talc [8]. Owing to starch low cost, availability, renewability, and biodegradability, PLA/starch (PSt) blends have been studied extensively for packaging materials. However, the addition of starch increased PLA’s tensile strength, but its flexibility was reduced [9]. The effect of starch on PLA’s mechanical properties is mainly due to starch’s hydrophilicity and PLA’s hydrophobicity, resulting in incompatibility between the two polymers. Therefore, compatibilizer [10] and plasticizer [11] can be added into polymer to improve the compatibility between PLA and starch.

Plasticizer derived from epoxidized vegetable oils (EVO), such as epoxidized soybean oil and epoxidized palm oil (EPO), attract much attention due to their non-toxicity, abundantly found in nature, inexpensive, high viscosity, and biodegradable [12]. Ali et al. studied the mechanical properties of plasticized polylactic acid/epoxidized soybean oil (PLA/ESO) blend and reported a decrease in glass transition temperature (*T_g_*) with an increment of cold crystallization temperature and elongation at break of plasticized PLA [11]. Emad et al. interestingly have reported that the plasticization of PLA by EPO had improved the thermal and mechanical properties of PLA [13].

Thus, the current study aims to bridge the gap between properties of PLA and commodity plastic in the packing industry by tailoring the PLA’s properties. PLA was blended with starch and EPO as filler and plasticizer, respectively, through the solution casting technique. The effects of EPO at 5, 10, 15, and 20 wt % on PLA/starch blend on thermal, mechanical, morphological, structural properties, and soil burial tests were investigated and discussed.

## 2. Materials and Methods

### 2.1. Materials

PLA (Ingeo^TM^ biopolymer 3251D) was purchased from NatureWorks LLC, Minnetonka, MN, USA. Tapioca starch was acquired from Tan Ban Huat Sdn Bhd, Georgetown, Pulau Pinang, Malaysia. Epoxidized palm oil (density of 0.886 g/mL with oxirane oxygen content of 2.84) was obtained from Budi Oil Enterprise Sdn Bhd, Telok Gong, Port Klang, Selangor, Malaysia. Soil was used to test the degradation rate of the composites. Chloroform was obtained from HmbG chemicals (analytical grades).

### 2.2. Blend Preparation

PLA, tapioca starch and epoxidized palm oil blends (PSE) were mixed through the solution casting technique while using chloroform as the solvent. Firstly, PLA and starch were dried for 24 h at 40 °C in an air circulating oven. Secondly, PLA was dissolved in chloroform and stirred for 12 h. Starch was mixed with chloroform before being added to dissolved PLA solution to avoid aggregation, followed by the addition of EPO (*w*/*w*) in the liquid form. The PLA/EPO/starch blend was mixed well for 24 h in a fume hood. The solution was poured into a petri dish and left to dry in a fume hood for three days. The blend ratios of PLA, starch, and EPO are listed in Table 1. After the drying process, the film was crushed and molded into 3 mm thickness sheet under a pressure of 44.482 kilonewton (kN) at 180 °C for 10 min. The film sheet was then used for further analysis.

### 2.3. Materials Characterizations

Thermal analysis was carried out using Differential Scanning Calorimetry (DSC 822, Mettler Toledo, Shah Alam, Selangor, Malaysia) to determine the glass transition temperature (*T_g_*), melting temperature (*T_m_*), and the cold crystallization temperature (*T_cc_*) of the composites. The analysis was set from 25 °C to 180 °C at a rate of 10 °C/min. Data from the second run was used for further analysis as the first run from DSC was to remove the thermal history of the blends. Thermal stability of neat PLA, PSt, and PSE10 blends were analyzed by Thermogravimetric Analyzer (Q500 series, TA Instruments Inc., Petaling Jaya, Selangor, Malaysia) heated from 25 to 400 °C at the rate of 10 °C/min under nitrogen atmosphere (20 mL/min). The onset degradation temperature *T*_0_, the maximum degradation temperature *T_max_*, and the degradation temperature referring to the percentage of material remaining 50% (*T*_50_) and 95% (*T*_95_) were evaluated. The yield strength, Young’s modulus, and elongation-at-break of polymers were determined while using Universal Testing Machine (AGS-X, Shimadzu, Petaling Jaya, Selangor, Malaysia). Samples for tensile testing were prepared according to ASTM D-638 type-V with crosshead speed of 5 mm/min. Five specimens for each blend were prepared and tested for mechanical tests. Charpy impact test was performed using Dynisco, Dynisco Polymer Test, Simatic OP7 machine, Seri Manjung, Perak, Malaysia with pendulum weight of 0.98 kg to characterize the impact resistance properties of un-notched PSE samples prepared according to ASTM D256. The functional groups in the PSt and PSE composites were examined while using Fourier Transform Infrared (FTIR-8400 Spectrophotometer, SHIMADZU Malaysia Sdn Bhd, Petaling Jaya, Selangor, Malaysia) with attenuated reflectance. Sixteen scans were conducted and spectra in absorbance mode between 4000 and 400 cm^−1^ wavenumber range at 4 cm resolution were collected. The surface morphology of neat PLA, PSt, and PSE10 blends were examined using scanning electronic microscope (JSM-5600, JEOL, Tokyo, Japan) with 10 kV accelerating voltage. The fractured surface of tensile test samples was coated with a thin layer of gold and mounted in an aluminum pan with carbon tape prior to the analysis. Samples were observed at 50 and 100 μm aperture. For soil burial test, all of the blend films (size of 20 mm × 20 mm × 3 mm) were buried in the plastic boxes containing soil at the laboratory. The relative humidity was maintained approximately at 40% and carried out at room temperature. The degradation of specimen was monitored and data was collected monthly for 5 months period. The samples were washed with deionized water and dried in an oven overnight at 40 °C before the weight loss degeneration was measured as a function of time. The biodegradation rate was measured while using the following equation:

(1) $$\mathrm{Biodegradation}\;\mathrm{rate}\;(\%)=\frac{W_{0}-W_{1}}{W_{0}}\times 100$$

where *W*_0_ is the original weight of the sample before biodegradation test and w_1_ is sample weight after biodegradation.

## 3. Results and Discussion

### 3.1. Thermal Properties

**Figure 1:**
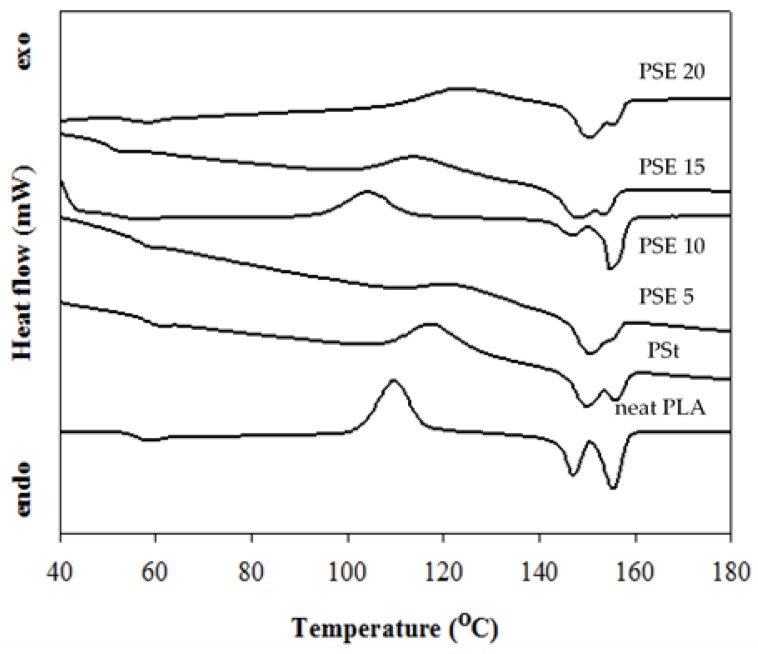
Differential scanning calorimetry (DSC) thermograms of neat PLA, PLA/starch (PSt), and PSE blends.

Thermal properties of neat PLA, PSt, and PSE blends with various EPO loadings are shown in Figure 1 and summarized in Table 2. The incorporation of starch into PLA matrix (PSt) caused a slight decreased in PLA′s *T_g_*. This could be due to moisture presence in starch; therefore, it acted as the starch plasticizer [14]. The addition of EPO into PSt shifted the *T_g_* of PLA to the lower temperature. The *T_g_* decreased from 58.5 °C for PSt to 57.5, 52.5, 55.4, and 56.1 °C for PSE5, 10, 15, and 20, respectively. This denoted to the plasticizing effect of EPO, where EPO penetrates into PLA chains promoting the segment mobility as a result of high surface contact between PLA/starch matrix and EPO during the solution casting process [15]. However, as EPO content increased above 10 wt %, *T_g_* started to increase and this could be due to reduced effect of EPO as plasticizer at higher amount. As for the cold crystallization, solution casting of starch and neat PLA blend increased *T_cc_* from 109.1 °C (neat PLA) to 118.3 °C (PSt). Upon the addition of EPO, the *T_cc_* of PSt dropped from 118.3 to 110, 108, and 115.1 °C at 10, 15 and 20 wt % EPO contents, while *T_cc_* was observed at the higher temperature (124.1 °C) for PSE5. For the melting behavior, two endothermic peaks (*T_m_*_1_, *T_m_*_2_) for neat PLA, PSt, and PSE blends ranging between 147–151.5 °C were observed. The double endothermic peaks of neat PLA and its composites blend have been reported due to PLA’s slow crystallization rate [16]. The ∆*H_m_* of all blends decreased when compared to PLA could attribute to the increases of polymer chain mobility [17].

### 3.2. Thermogravimetric Analysis

**Figure 2:**
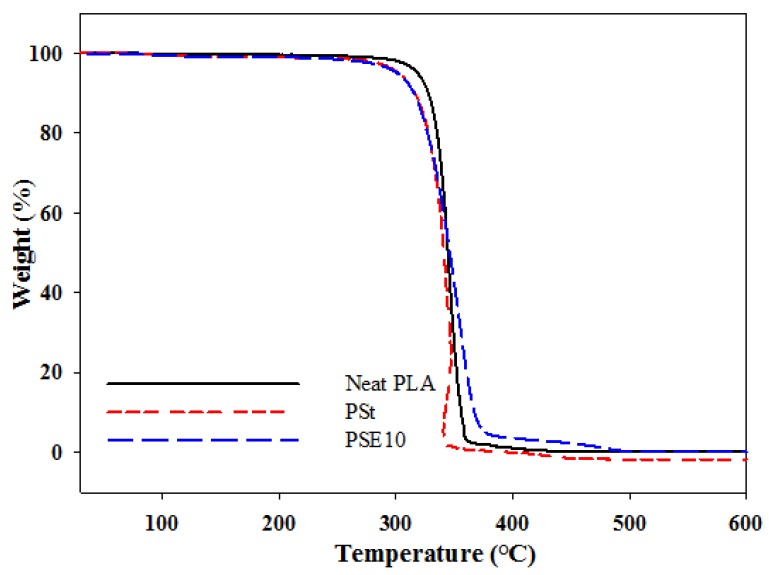
Thermogravimetric (TGA) thermograms of neat PLA, PSt, and PSE10.

The weight loss of neat PLA, PSt, and PSE10 as a function of temperature is shown in Figure 2. The onset degradation temperature (*T*_0_), maximum degradation temperature (*T_max_*), and the degradation temperature referring to the percentage of material remaining, 50% (*T*_50_), 95% (*T*_95_), and char formation are tabulated in Table 3. All of the blends showed thermal stability below 290 °C. However, the incorporation of starch into neat PLA matrix resulted in the onset degradation temperature occuring at lower temperature (268 °C) than neat PLA (295 °C). Similar thermal degradation behavior in PLA/starch was also reported by Guan [18]. From the thermogravimetric analysis (TGA) curves, the difference between PSt and PSE10 curve was hardly differentiated mainly because starch degradation and PLA hydrolysis occurred concurrently. However, above 300 °C the degradation of PSE10 was slower when compared to PSt. From Table 3, the thermal stability of PSE10 increased at *T_max_*, *T*_50_ and *T*_95_ by 38, 5, and 36 °C, respectively, when compared to PSt. As the temperature increased, PSE10 showed higher ability to resist thermal degradation as compared to PSt blend. The higher thermal stability of PSE10 is due to char formation. The presence of EPO formed a shield retardant of oxidization and prevented volatile products being released from the PSt matrix [19].

### 3.3. Mechanical Properties

#### 3.3.1. Young’s Modulus

**Figure 3:**
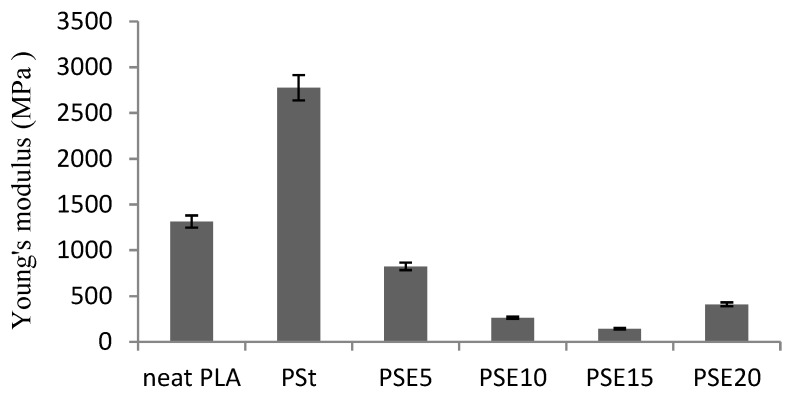
Young’s modulus of neat PLA, PSt, and PSE blends.

From Figure 3, the plasticization of PSt blend with EPO at all loads reduced Young’s modulus rapidly, indicating an increase in free volume created by EPO plasticizing effect that results in a higher flexibility of PLA chains. However, it can be noticed that the Young’s modulus of PSE20 blend increased slightly. According to Silverajah et al., at a higher EPO content, the PLA matrix would be saturated and resulted in excess EPO accumulating at the interphase area where plasticizer–plasticizer interaction is favored [20].

#### 3.3.2. Yield Strength

**Figure 4:**
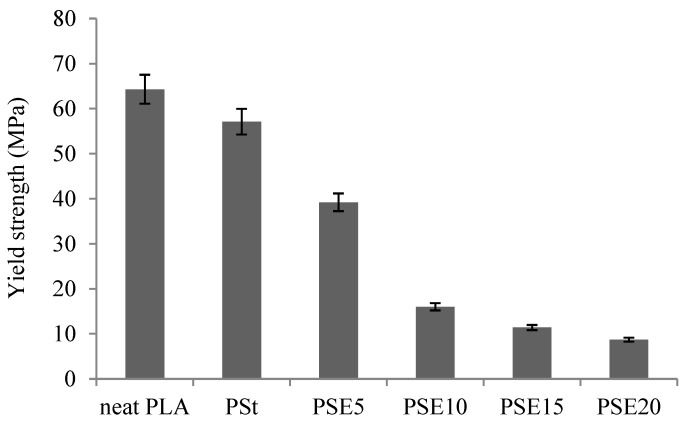
Yield strength of neat PLA, PSt, and PSE blends.

The yield strength decreased with the addition of EPO to PSt blends by 31.3%, 79.1%, 80%, and 84.8% for EPO content of 5, 10, 15, and 20 wt %, respectively (Figure 4). The drop in yield strength could be due to the poor cohesion between neat PLA (hydrophobic), starch (hydrophilic), and EPO (hydrophobic). It was observed that 5 wt % of EPO had the minimum effect on the yield strength of PSt, which may indicate that 5 wt % was sufficient to act as the lubricant for PLA chain creating a free volume which allowed a better dispersion of EPO in PSt matrix and higher interaction at the interfacial area of the blend [21]. In addition, phase separation occurred as EPO content increased, which resulted in the decline of plasticized PSt blend’s yield strength. This result could be attributed to plasticizer–plasticizer interaction dominating at interfacial phase, which affected the homogeneity of EPO dispersion in PSt blend [20].

#### 3.3.3. Elongation-at-Break

**Figure 5:**
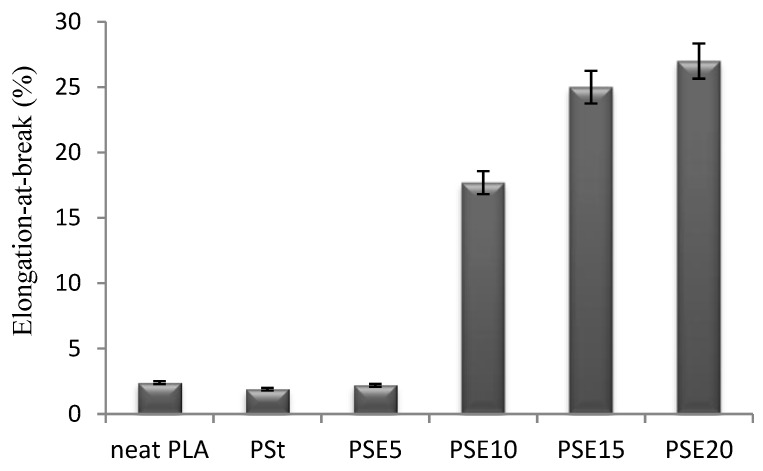
Elongation-at-break of neat PLA, PSt, and PSE blends.

The elongation-at-break of PSE5, PSE10, PSE15, and PSE20 were increased by 15.7%, 831.5%, 1215.8%, and 1321.07%, respectively, with the content of EPO (Figure 5). The increase in the elongation-at-break of plasticized PSE blends denoted the increase in blend’s ductility. This is due to the plasticizing effect of EPO dispersing into PLA matrix resulting in intermolecular interaction between EPO and PLA causing an increment in the chain mobility.

#### 3.3.4. Impact Test

**Figure 6:**
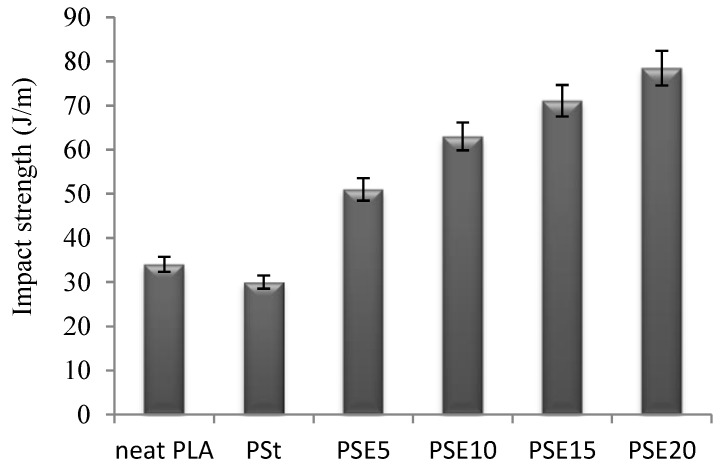
Impact strength of neat PLA, PSt, and PSE blends.

The impact resistances of neat PLA, PSt, and PSE blends are illustrated in Figure 6. The presence of EPO in PLA/starch blends dramatically increased the impact strength of PSt 30 J/m by 70% (PSE5), 110% (PSE10), 137% (PSE15), and 161.6% (PSE20) indicated an improvement in the interfacial adhesion compatibility between composites. PSE20 recorded the highest impact strength, indicating a higher amount of EPO penetrates into PLA matrix, resulting in a better homogeneity and acting as energy distributor at the point of impact.

#### 3.3.5. Dynamic Mechanical Analyzer (DMA)

**Figure 7:**
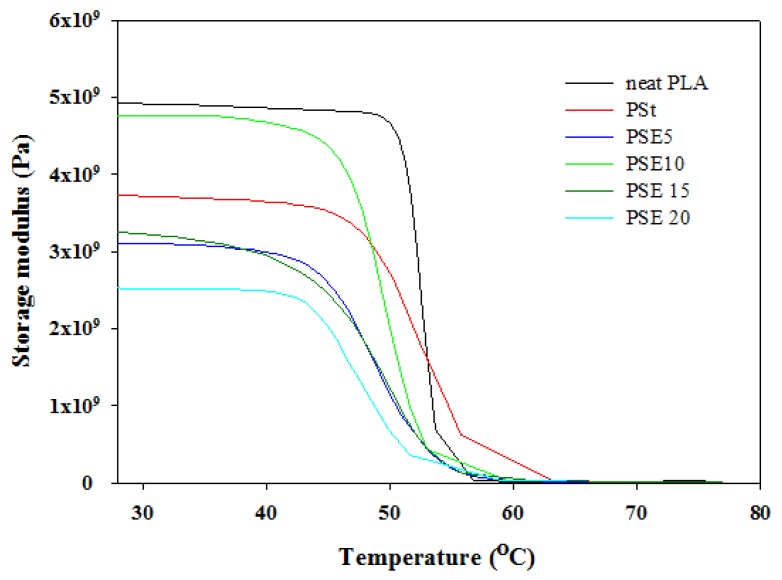
Storage modulus of neat PLA, PSt, and PSE blends.

**Figure 8:**
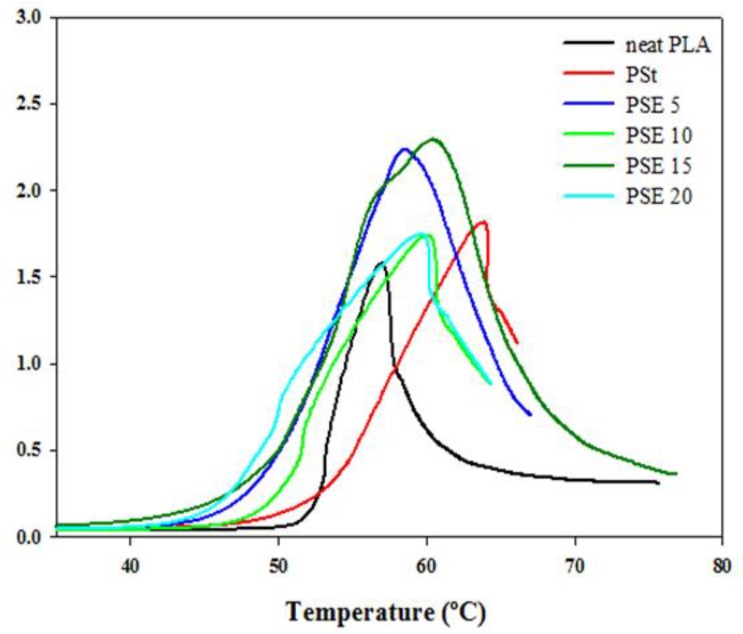
Tan δ of neat PLA, PSt, and PSE blends.

The storage modulus and Tan δ as a function of temperature are shown in Figure 7 and Figure 8, respectively. In all blends, including neat PLA, the storage moduli remained constant in the glassy region until it reached the *T_g_* (45–54 °C). It can be noticed that the addition of starch significantly decreased the storage moduli, and it was further decreased as plasticizer was added. The storage modulus of PSt blend reduced when compared to neat PLA could be due to moisture presence in starch, therefore it acted as the starch plasticizer [14]. Plasticization of PLA/starch with EPO at 5, 10, 15, and 20 wt % reduced the storage modulus, suggesting an increase in the chain mobility of neat PLA as a result of the increase in amorphous region mobility and interphase relaxation. As presented in Table 4, starch decreased the *T_g_* of neat PLA from 64.2 °C to 62.4 °C, which indicates that moisture presence in starch acted as plasticizer. However, the *T_g_* of PSt (62.4 °C) shifted downward by 2.2 °C (PSE5), 2.3 °C (PSE10), and 0.2 °C (PSE15), while it increased by 0.7 °C (PSE20). The variations of *T_g_* obtained from DSC and dynamic mechanical analyzer (DMA) are due to different thermal measurement methods.

### 3.4. Fourier Transform Infrared Spectroscopy (FTIR)

**Figure 9:**
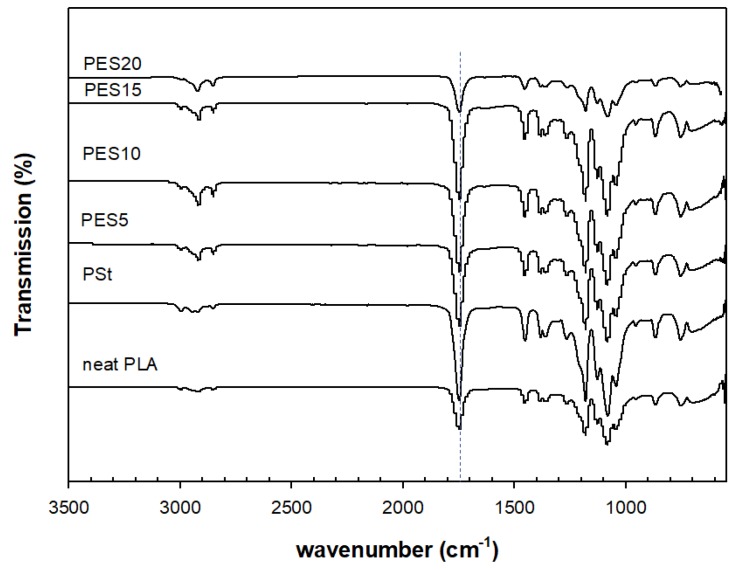
Fourier transform infrared spectroscopy (FTIR) spectrum of neat PLA, PSt, and PSE blends.

In Figure 9, the distinctive characteristics transmittance bands of neat PLA, PSt, and PSE blends’ occurred at 2800–3000 cm^−1^ (–CH_2_ stretch), 1450–1453 cm^−1^ (–CH_3_ bending), and 1381cm^−1^ (C–H bending). It also depicts a clear stretch of carbonyl group (C=O) of ester bond around 1747 cm^−1^ along with symmetrical –C–O–C stretch at 1180 cm^−1^, and asymmetrical –CH_3_ stretch in 1080 cm^−1^. The carbonyl group of the aliphatic ester in the PSE blends remains at 1747 cm^−1^.

### 3.5. Surface Morphology

**Figure 10:**
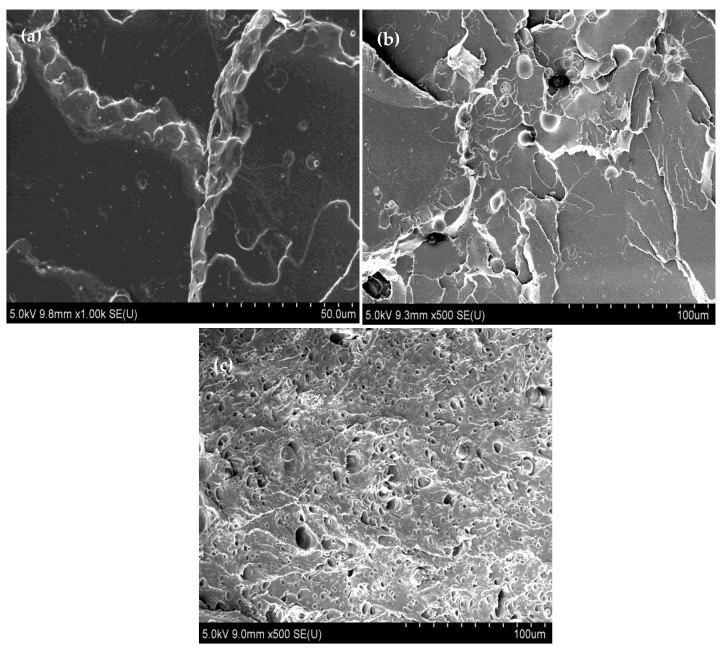
Surface morphology of (**a**) neat PLA, (**b**) PSt and (**c**) PSE 10.

The surface morphology of fracture surfaces of neat PLA, PSt, and PSE10 after failed tensile testing are presented in Figure 10. PSE10 was selected to observe the surface morphology based on the mechanical testing result. PLA exhibited smooth and homogeneous surface, indicating its brittle behavior (Figure 10a). After blending 5 wt % of tapioca starch with neat PLA (Figure 10b), the surface was less homogeneous and this could be due to the incompatibility between starch and PLA. A ductile fractured surface of PSE10 can be observed in Figure 10c, which illustrates an elongated microvoid with white fibrils scattered throughout the fractured surface of strained PSE10. These dark microvoids occurred as EPO microdroplets accumulated within PLA matrix surface forming EPO rich phase. Similar morphological behaviors of plasticized PLA/starch have also been reported [22,23].

### 3.6. Soil Burial Test

**Figure 11:**
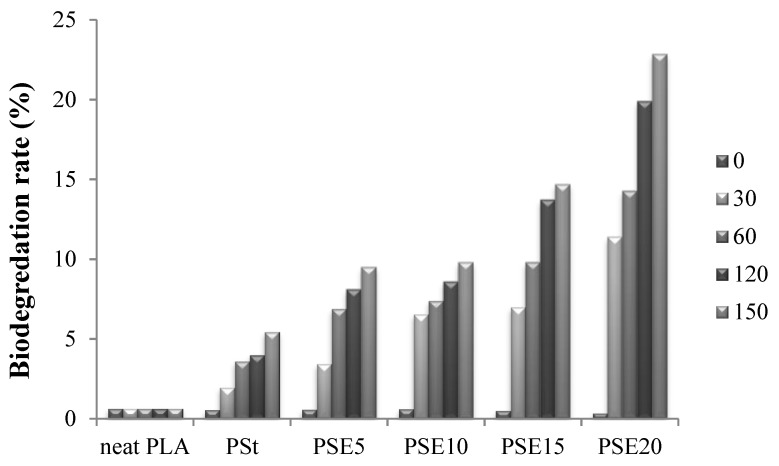
Soil burial test of neat PLA, PSt, and PSE blends.

Biodegradation tests were conducted using soil burial test. The weight loss of neat PLA, PSt, and PSE blends samples as a function of time in soil is demonstrated in Figure 11. Neat PLA sample weight remained constant and no physical changes were observed through the five months of testing period. The rate of neat PLA degradation was increased to 2% by the incorporation of 5 wt % of starch. As EPO contents increase, the biodegradation rate of PSt blend increased over 150 days by 4.01, 4.35, 9.25, and 17.35 % for EPO content 5, 10, 15, and 20 wt %, respectively. This could be due to the release of EPO, which is low molecular weight plasticizer from the polymer matrix. The free volume created by plasticizer in the blend matrix could facilitate the moisture to access deeper into PLA matrix accelerating the hydrolysis of PLA chain [24].

## 4. Conclusions

The effect of EPO at 5, 10, 15, and 20 wt % content on thermal, mechanical, structural, morphological, and biodegradability of solution casted PSE blends were investigated. The blends were prepared through the solution casting technique and then poured onto petri dishes. The blend films were molded into film sheets for further analysis. From the thermal analysis, PSE blends had lower *T_g_* when compared to PSt, indicating an increase in chain mobility of the plasticized PLA. The cold crystallization temperature of PSt decreased as EPO contents increases. Meanwhile, melting temperature of PSt decreased at all EPO contents. The thermal stability of PSE blends increased the blends ability to resist thermal degradation. The mechanical properties of plasticized PSt improved Young’s modulus, yield strength, elongation-at-breaks, and impact strength attributed to uniform dispersion of EPO in PLA matrix. Storage modulus decreased and Tan δ increased as EPO contents increased because of the increase in elastic response of PSE blends. Although effect of EPO could be observed by the thermal and mechanical analysis, PSE blends spectra showed C=O of the aliphatic ester and C–O in –CH–O– at 1748 and 1182 cm^−1^ as the PLA. Surface morphologies of tensile failed samples demonstrated ductile surface for PSE blends when compared to brittle surface of PSt. The biodegradability of PSE blends increased with increasing amount of EPO in the PLA/starch.

## Figures and Tables

**Table 1:** Composition and ratio of polylactic acid (PLA), starch, and epoxidized palm oil (EPO) in the PLA/starch/EPO (PSE) blend.

Blend ID	Composition Ratio (wt %)		
	PLA	Starch	EPO
Neat PLA	100	0	0
PLA/starch (PSt)	95	5	
PSE5	90	5	5
PSE10	85	5	10
PSE15	80	5	15
PSE20	75	5	20

**Table 2:** Thermal Properties of neat PLA, PSt, and PSE blends.

Blend ID	*T_g_* (°C)	*T_cc_* (°C)	∆*H_cc_* (J/g)	*T_m1_* (°C)	*T_m2_* (°C)	∆*H_m_* (J/g)
neat PLA	59.1	109.1	37	148.0	156	22
PSt	58.5	118.3	38	151.5	157	10
PSE5	57.5	124.1	12	151.0	156	10
PSE10	52.5	110.0	12	147.1	155	5.5
PSE15	55.4	109.0	25	150.1	155	11
PSE20	56.1	115.1	30	151.2	155	12

**Table 3:** Thermal degradation temperatures of neat PLA, PSt, and PSE10.

Samples	*T_g_* (°C)	*T_max_* (°C)	*T*_50_ (°C)	*T*_95_ (°C)
Neat PLA	395	360	344	358
PSt	268	346	341	340
PSE10	268	384	346	376

**Table 4:** Tan δ and *T_g_* of neat PLA, PSt, and PSE blends obtained from dynamic mechanical analyzer (DMA).

Samples	*T_g_* (°C)	Tan δ
Neat PLA	64.2	1.043
PSt	62.4	1.76
PSE 5	60.2	2.37
PSE 10	60.1	1.16
PSE 15	62.1	2.12
PSE 20	63.1	2.37
